# Dynamics of superparamagnetic nanoparticles in viscous liquids in rotating magnetic fields

**DOI:** 10.3762/bjnano.10.221

**Published:** 2019-11-22

**Authors:** Nikolai A Usov, Ruslan A Rytov, Vasiliy A Bautin

**Affiliations:** 1 National University of Science and Technology «MISIS», 119049, Moscow, Russia; 2 Pushkov Institute of Terrestrial Magnetism, Ionosphere and Radio Wave Propagation, Russian Academy of Sciences, IZMIRAN, 108480, Troitsk, Moscow, Russia

**Keywords:** magnetic hyperthermia, magnetic nanoparticles, numerical simulation, rotating magnetic field, specific absorption rate, viscous liquid

## Abstract

The dynamics of magnetic nanoparticles in a viscous liquid in a rotating magnetic field has been studied by means of numerical simulations and analytical calculations. In the magneto-dynamics approximation three different modes of motion of the unit magnetization vector and particle director are distinguished depending on frequency and amplitude of the rotating magnetic field. The specific absorption rate of a dilute assembly of superparamagnetic nanoparticles in rotating magnetic field is calculated by solving the Landau–Lifshitz stochastic equation for the unit magnetization vector and the stochastic equation for the particle director. At elevated frequencies an optimal range of particle diameters is found where the specific absorption rate of an assembly in a rotating magnetic field has a maximum. It is shown that with an optimal choice of the particle sizes sufficiently large SAR values of the order of 400–500 W/g can be obtained in a rotating magnetic field with a frequency *f* = 400 kHz and a moderate magnetic field amplitude *H*_0_ = 100 Oe.

## Introduction

Magnetic nanoparticles are promising materials in various areas of biomedicine [1–4], such as magnetic resonance imaging [5–7], targeted drug delivery [8–10], and magnetic hyperthermia [11–20]. Iron oxide nanoparticles are most frequently used in biomedicine due to their biocompatibility, biodegradability and relatively high saturation magnetization. In magnetic hyperthermia [2–3,11–20] magnetic nanoparticles are directly introduced into a tumor and are exposed to an alternating magnetic field (AMF) of frequency *f* = 100–500 kHz and amplitude *H*_0_ = 100–200 Oe. This would allow the tumor temperature to be maintained at about 42 °C if a magnetic nanoparticle assembly were capable to absorb efficiently the energy of the alternating magnetic field. According to a number of medical indications [1,3,19–20], certain thermal effects in combination with radiotherapy or chemotherapy can significantly improve the results of cancer treatment.

One of the main technological problems of current magnetic hyperthermia development is the optimal choice of sizes and magnetic parameters of nanoparticles, as well as the selection of appropriate AMF frequency and amplitude. Besides, there are different biological environments of an assembly of magnetic nanoparticles in the human body [1–3]. In most cases magnetic nanoparticles penetrate directly into the tumor cells or surrounding tissues [2–3]. Inside the cells magnetic nanoparticles usually form dense clusters tightly bound to the surrounding tissues [21–24], so that the rotation of a nanoparticle as a whole in AMF is difficult or completely absent. Thus, the AMF energy absorption is only associated with the dynamics of the magnetic moments of the particle. However, if nanoparticles remain distributed in biological fluids (blood, serum), the intensity of AMF energy absorption is determined also by the rotation of the nanoparticles as a whole in a viscous liquid [25–26].

Various mathematical approaches are necessary for a theoretical description of the energy absorption processes for assemblies of immobilized and freely rotating nanoparticles. In dense nanoparticle assemblies that are tightly bound to surrounding tissues the mechanical rotation of the particles is inhibited. However, one has to take into account the influence of strong magnetic dipole interaction between nanoparticles [27–32] on the energy absorption intensity. On the other hand, for particles distributed in a viscous liquid it is necessary to take into account [25] a coupled motion of the unit magnetization vector 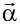 and the nanoparticle director 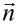 that is parallel to the direction of the easy anisotropy axis of a rotating nanoparticle.

Recently, the application of a rotating magnetic field (RMF) in biomedicine, in particular in magnetic hyperthermia, has been studied both theoretically [33–39] and experimentally [40–43]. Unfortunately, the specific absorption rate (SAR) measured in RMFs [41,43] for assemblies of particles distributed in a viscous liquid turned out to be very small, of the order of a few watts per gram of magnetic material. At the same time, the SAR of an assembly of superparamagnetic nanoparticles in AMF under the optimal conditions reaches values of the order of several hundred watts per gram [3,15–18]. It seems probable that the geometric and magnetic parameters of the particles used in the RMF experiments [41,43] were far from optimal. Therefore, it is important to determine the optimal geometric and magnetic parameters of the nanoparticles, as well as the amplitudes and frequencies at which the SAR of the superparamagnetic nanoparticle assembly in RMFs will be large enough to be used in magnetic hyperthermia.

In this work, detailed numerical calculations of the SAR in RMFs for a dilute assembly of superparamagnetic particles with uniaxial anisotropy distributed in a viscous liquid have been carried out. First, the behavior of a magnetic particle in a RMF is studied in the magneto-dynamics approximation [25,44–45] neglecting the thermal fluctuations of the particle magnetic moment and the particle director. On the plane of parameters (*f*, *H*_0_) three domains for different modes of motion of the unit magnetization vector and particle director are distinguished. The boundaries between these domains, first determined numerically, are then confirmed by analytical calculations.

Then, the SAR of a dilute assembly of superparamagnetic nanoparticles in RMFs is calculated by solving the Landau–Lifshitz stochastic equation for the unit magnetization vector and the stochastic equation for the particle director. It is shown that at elevated frequencies, *f* > 100 kHz, there is an optimal range of particle diameters where the SAR in RMFs has a maximum. This behavior of the SAR in RMFs resembles the one in AMFs, [11,25]. For iron oxide nanoparticles of optimal diameter the SAR in RMFs reaches values of the order of 400–450 W/g at a frequency *f* = 400 kHz and moderate amplitude *H*_0_ = 100 Oe. It is important to note that for sufficiently large particle diameters the SAR in RMFs is approximately two times larger than that in AMFs.

It is worth mentioning that liquids with suspended particles of average size of the order of or less than 100 nm belong to the interesting class of nanofluids [46–47] or fluids with microstructure [48] that have received great attention recently due to their unique magnetohydrodynamic and heat-conduction properties. In this paper we consider the limit of a dilute assembly of magnetic nanoparticles in a liquid, which is of particular interest and demonstrates the complex behavior of individual magnetic nanoparticles in a viscous liquid.

## Magneto-Dynamics Approximation

Let us consider first the dynamics in a viscous liquid of a spherical single-domain nanoparticle of a sufficiently large diameter, close to that of a single domain. In this case one can neglect the influence of thermal fluctuations on the behavior of magnetic moment and the particle director and describe their movement in RMFs in the magneto-dynamics approximation [25,44–45]. Without loss of generality one can assume that the magnetic field of constant frequency *f* and amplitude *H*_0_ rotates in the *XY*-plane of the Cartesian coordinates, so that

[1]
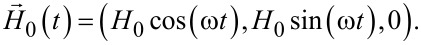


Neglecting weak magnetic damping and a small moment of inertia of a magnetic nanoparticle, the magneto-dynamic equations of motion of the unit vectors 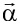 and 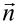 in a viscous fluid have the form [25]

[2]
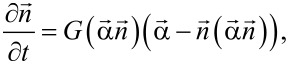


[3]
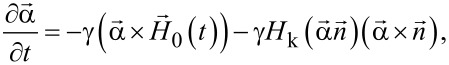


where *G* = *K*/3η, η is the liquid viscosity, *K* is the effective magnetic anisotropy constant of the nanoparticle, *H*_k_ = 2*K*/*M*_s_ is the particle anisotropy field, and *M*_s_ is the saturation magnetization.

**Figure 1:**
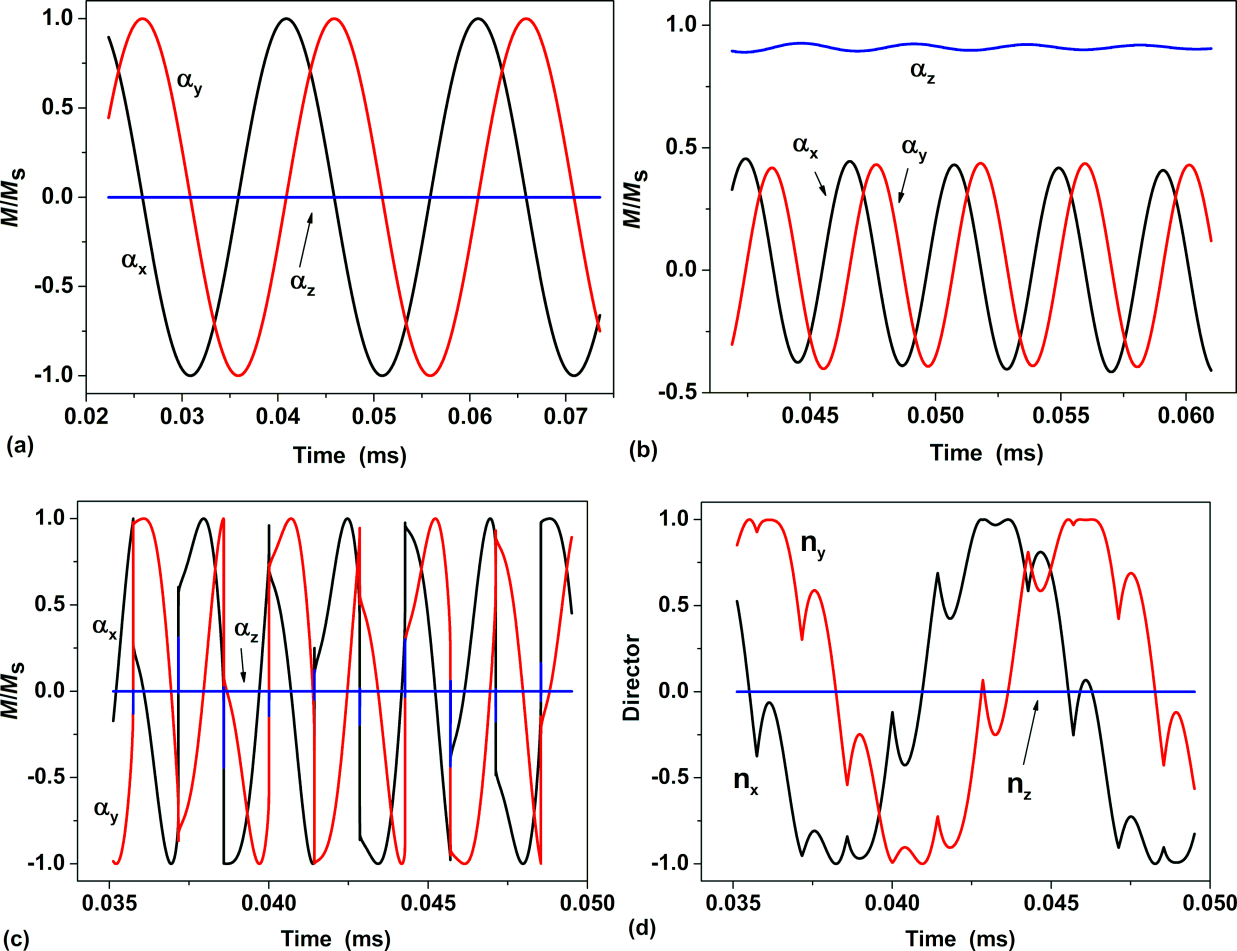
Dynamics of the unit magnetization vector in RMFs in the magneto-dynamics approximation for various regimes of stationary motion of a nanoparticle in a viscous liquid: a) first mode, *f* = 50 kHz, *H*_0_ = 200 Oe; b) second mode, *f* = 240 kHz, *H*_0_ = 100 Oe; c, d) particle dynamics in the third mode, *f* = 450 kHz, *H*_0_ = 400 Oe.

Equations 1–3 describe the complex coupled dynamics of the unit vectors 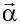 and 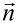 in RMFs. Numerical solution of Equations 1–3 with a small time step following the procedure described earlier [25] reveals three stationary modes of motion of the vectors 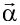 and 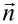 depending on frequency and amplitude of the RMF. Figure 1a,b show the regular dynamics of the vector 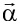 in the first and second modes of particle motion, respectively. The particle director moves in these modes in a similar way, but it has a constant time shift with respect to the vector 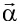. The dynamics of the vectors 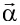 and 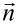 in the third mode of particle motion is shown in Figure 1c and Figure 1d, respectively. The illustrative calculations were performed for magnetic nanoparticles of iron oxide with a saturation magnetization *M*_s_ = 350 emu/cm^3^ and a magnetic anisotropy constant *K* = 10^5^ erg/cm^3^. The liquid viscosity is assumed to be η = 0.01 g/(cm·s).

**Figure 2:**
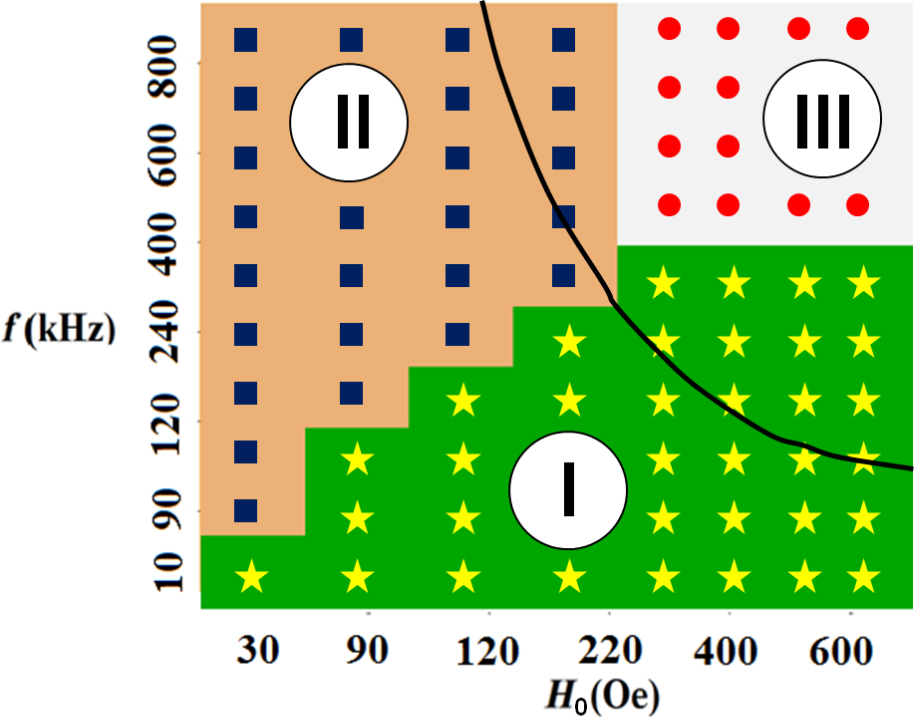
The domains of various magneto-dynamic modes of motion of a superparamagnetic nanoparticle in a viscous liquid depending on frequency and amplitude of the RMF. The Brezovich area [49–50] recommended for medical reasons for use in magnetic hyperthermia, *fH*_0_ ≤ 6.25 × 10^4^ kHz·Oe, is located below the black curve.

The domains of existence of various magneto-dynamic regimes I–III on the plane (*f*, *H*_0_) determined numerically using the abovementioned physical parameters are shown in Figure 2. Different symbols in this figure show the specific pairs of the parameters (*f*, *H*_0_) for which numerical calculations were performed. The area below the black curve in Figure 2 corresponds to the condition *fH*_0_ ≤ 6.25 × 10^4^ kHz·Oe. This domain of applied magnetic field frequencies and amplitudes is recommended for medical reasons to be used in magnetic hyperthermia [49–50].

In the first mode existing in domain I in Figure 2 at low and moderate RMF frequencies the vectors 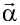 and 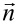 rotate in unison around the *Z*-axis with the RMF frequency. However, there are constant phase differences between the vectors 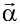, 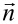, and also the magnetic field vector. At the same time, *Z*-components of the vectors 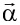 and 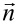 in the domain I are close to zero, so that the rotation of these vectors occurs in fact near the *XY*-plane. An example of such a motion for the unit magnetization vector 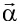 is shown in Figure 1a.

In the second mode existing in domain II in Figure 2 both vectors go out of the *XY*-plane. Actually, they have significant components parallel to the *Z*-axis. An example of motion of the unit magnetization vector in domain II is shown in Figure 1b. The vector 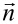 moves similarly in domain II. Projections of the vectors 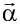 and 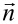 on the *XY*-plane show constant phase differences between both vectors and also the magnetic field vector.

Finally, in the third mode existing in domain III in Figure 2 the vectors 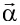 and 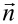 return to the plane of magnetic field rotation. However, they move in this plane with different average frequencies. The unit magnetization vector gradually lags behind the magnetic field vector and periodically jumps from one magnetic potential well to another. This behavior of the unit magnetization vector components is shown in Figure 1c. As Figure 1d shows, the director of the particle also rotates around the *Z*-axis with a reduced average frequency. When the vector 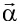 jumps it experiences complex oscillatory movements.

To confirm the features of the particle magneto-dynamics in RMFs obtained numerically, and to extend these results to a wider range of physical parameters we also carried out an analytical analysis of Equations 1–3 in the Appendix section. The analytical solution presented in the Appendix section describes the behavior of vectors 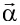 and 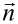 in the domains I and II on the plane of parameters (*f*, *H*_0_) shown in Figure 2.

**Figure 3:**
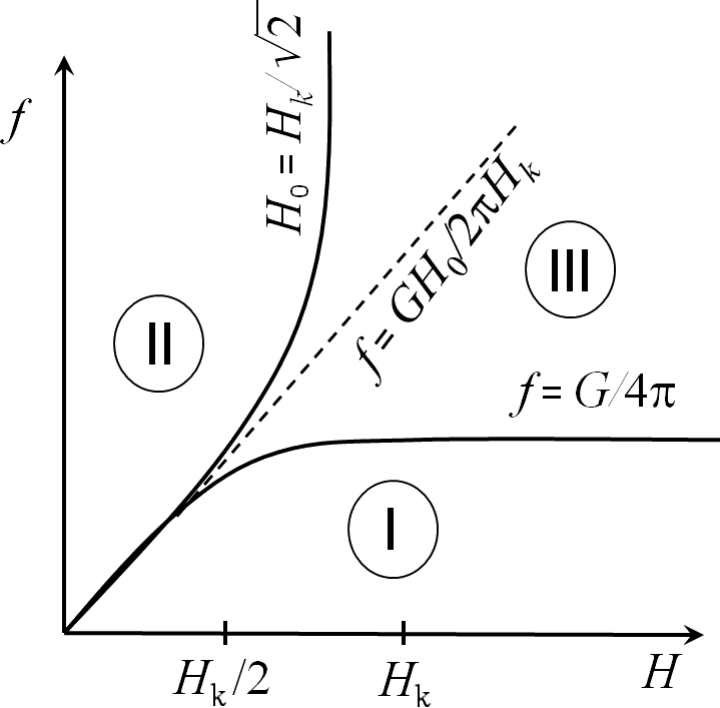
The domains I–III of the different magneto-dynamic modes of nanoparticle motion in a viscous liquid in RMFs obtained analytically based on Equations 9–11 (see Appendix section).

The boundaries between the domains I–III of various magneto-dynamic modes of particle motion in a viscous liquid in RMFs, obtained as a result of the analysis of the nonlinear system of equations investigated in the Appendix section, are shown in Figure 3. The obtained analytical results are in excellent agreement with the numerically defined regions shown in Figure 2 for specific values of *M*_s_, *K* and η.

## SAR in RMFs

We now turn to the SAR calculation for a dilute assembly of superparamagnetic nanoparticles in RMFs, taking into account thermal fluctuations of the magnetic moment and the director of a superparamagnetic nanoparticle. The SAR calculations were carried out by solving jointly the Landau–Lifshitz stochastic equation for the unit magnetization vector and the stochastic equation for the director of a superparamagnetic nanoparticle.

The stochastic Landau–Lifshitz equation for the unit magnetization vector of the particle has the form [51–54]

[4]
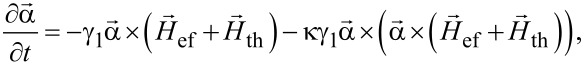


where γ_1_ = |γ|/(1 + κ^2^), κ is the phenomenological damping parameter, 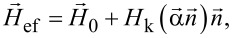 and 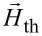 is the random thermal magnetic field that causes thermal fluctuations of the particle magnetic moment. The stochastic equation for the nanoparticle director is given by [25,54–55]

[5]
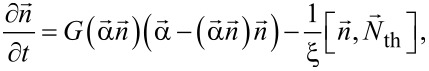


where ξ = 6η*V* is the friction coefficient of a particle in a viscous liquid, *V* is the particle volume, and 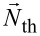 is the fluctuating rotational moment that describes the free Brownian rotational motion of a particle in a liquid in the absence of an external magnetic field.

In accordance with the fluctuation–dissipation theorem [54], the components of the fluctuating rotational moment satisfy the statistical relations [55], (*i,j* = *x*,*y*,*z*),

[6]
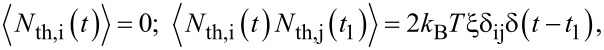


where *k*_B_ is the Boltzmann constant, *T* is the absolute temperature, δ_αβ_ is the Kronecker’s symbol, and δ(*t*) is the delta function. For the components of the fluctuating thermal magnetic field there are similar statistical relations [51]:

[7]
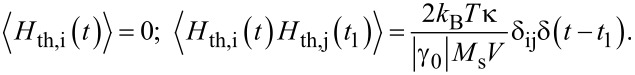


The SAR of a dilute assembly of superparamagnetic nanoparticles in magnetic field rotating at a frequency *f* in the *XY*-plane is determined by the integral

[8]
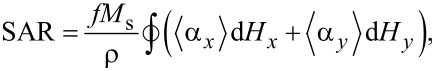


where ρ is the nanoparticle density. The averaged components of the unit magnetization vector, ⟨α*_x_*⟩ and ⟨α*_y_*⟩, are calculated by solving the stochastic Equations 4–7 and averaging the results over a sufficiently large number of independent numerical experiments carried out for the same magnetic nanoparticle under arbitrary initial conditions.

**Figure 4:**
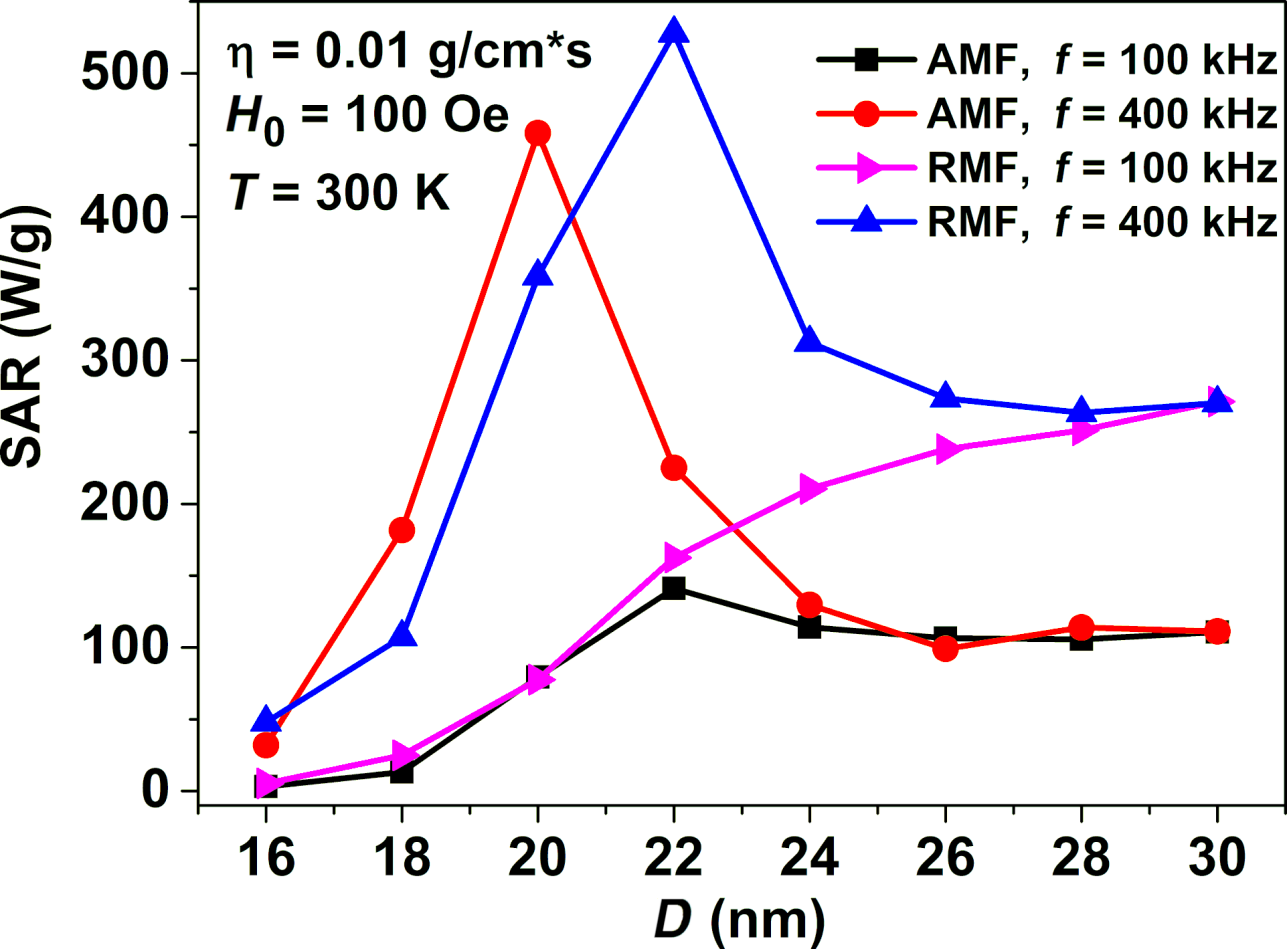
Comparison of the SAR of a dilute assembly of superparamagnetic nanoparticles in AMFs and RMFs depending on the nanoparticle diameter for two frequencies, *f* = 100 and 400 kHz, at a fixed magnetic field amplitude, *H*_0_ = 100 Oe.

First of all, it is interesting to compare the results of the SAR calculation of a dilute assembly of superparamagnetic nanoparticles distributed in a viscous fluid in RMFs and AMFs. In the calculations presented in Figure 4 the saturation magnetization of nanoparticles is given by *M*_s_ = 350 emu/cm^3^, the effective magnetic anisotropy constant *K* = 10^5^ erg/cm^3^, the particle density ρ = 5 g/cm^3^, and the viscosity of the liquid η = 0.01 g/(cm·s). The magnetic damping constant is assumed to be κ = 0.1, the medium temperature is *T* = 300 K.

Figure 4 shows that for a dilute assembly of superparamagnetic nanoparticles in RMFs the SAR value monotonously rises with increasing particle diameter at a moderate frequency *f* = 100 kHz. However, with an increase in the frequency to *f* = 400 kHz a rather narrow region of optimal nanoparticle diameters appears, *D* = 20–24 nm, in which the SAR reaches its maximum values. The behavior of SAR depending on the nanoparticle diameter in AMFs is similar. However, it is important to note that as Figure 4 shows in the range of particle diameters *D* > 24 nm the SAR in RMFs is approximately two times larger than that in AMFs.

**Figure 5:**
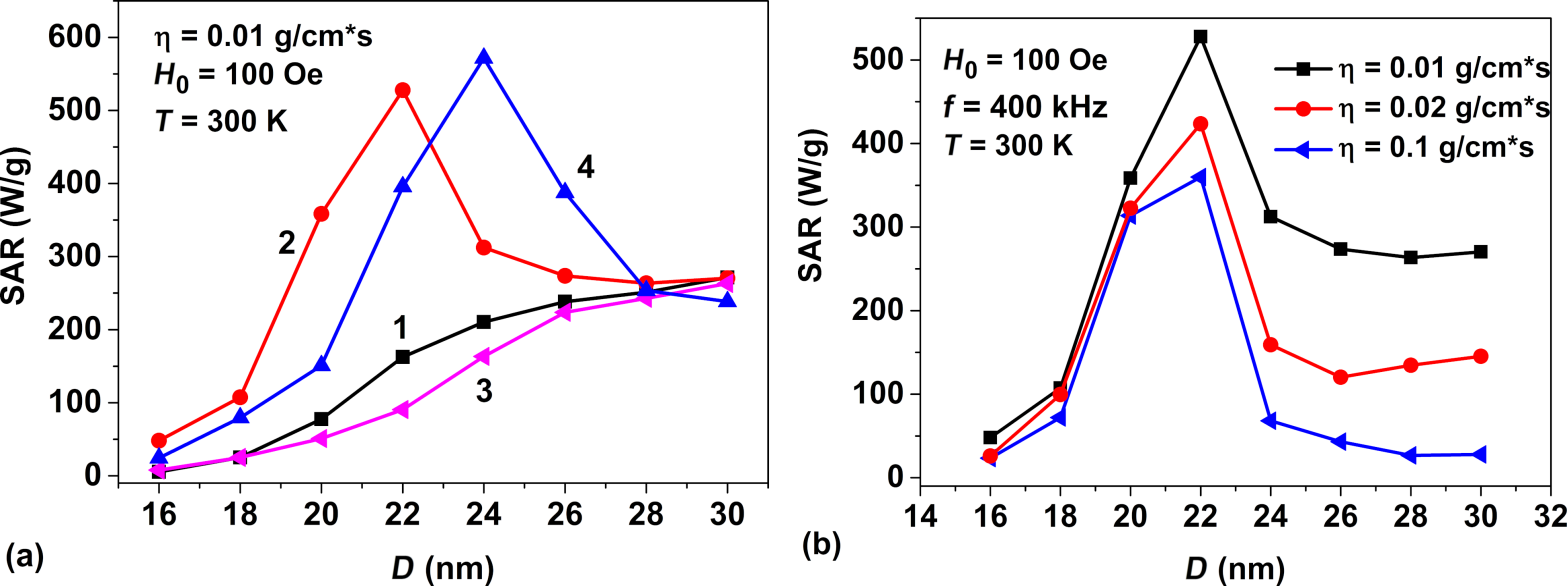
a) SAR in RMFs for dilute assemblies of superparamagnetic nanoparticles with various magnetic anisotropy constants: 1) *K* = 10^5^ erg/cm^3^, *f* = 100 kHz, 2) *K* = 10^5^ erg/cm^3^, *f* = 400 kHz, 3) *K* = 8 × 10^4^ erg/cm^3^, *f* = 100 kHz, 4) *K* = 8 × 10^4^ erg/cm^3^, *f* = 400 kHz. b) SAR of assemblies of superparamagnetic nanoparticles depending on the viscosity of the liquid.

For the sake of completeness, we also calculated the SAR in RMFs for assemblies of superparamagnetic nanoparticles with different magnetic anisotropy constants and in liquids of different viscosities. As Figure 5a shows, with a slight decrease in the magnetic anisotropy constant the dependence of the SAR on the average nanoparticle diameter does not change appreciably, whereas the SAR maximum shifts to larger particle diameters. Figure 5b shows the dependence of the SAR on the average diameter of nanoparticles in liquids of various viscosities. One can see in this figure that the range of optimal particle diameters varies little in the range of η = 0.01–0.1 g/(cm·s), but the SAR decreases with increasing viscosity, especially in the region of relatively large nanoparticle diameters.

## Results and Discussion

The results of numerical simulations presented in Figure 4 and Figure 5 show that with an optimal choice of the particle diameters sufficiently large SAR values, of the order of 400–500 W/g can be obtained in RMFs at a frequency *f* = 400 kHz and a moderate amplitude *H*_0_ = 100 Oe. Nevertheless, the experimentally measured [41,43] SAR values in RMFs for an assembly of iron oxide nanoparticles distributed in a viscous liquid turned out to be very small, only about 1.0–4.0 W/g. This may be due to the small RMF amplitudes used in the experiments [41,43]. Indeed, in [43] the SAR values of the assembly in RMFs were measured in a fairly wide frequency range, from 100 to 800 kHz. However, the RMF amplitudes were only 1 or 2 kA/m, that is, it did not exceed 25 Oe. As our numerical simulations show, it is impossible to obtain noticeable SAR values with such small RMF amplitudes. In [41] the SAR measurements were carried out at moderate frequencies, *f* = 130 and 160 kHz, but the RMF amplitude was higher, *H*_0_ = 4.1 kA/m. However, in this case the measured SAR values [41] also turned out to be small, of the order of 1 W/g.

**Figure 6:**
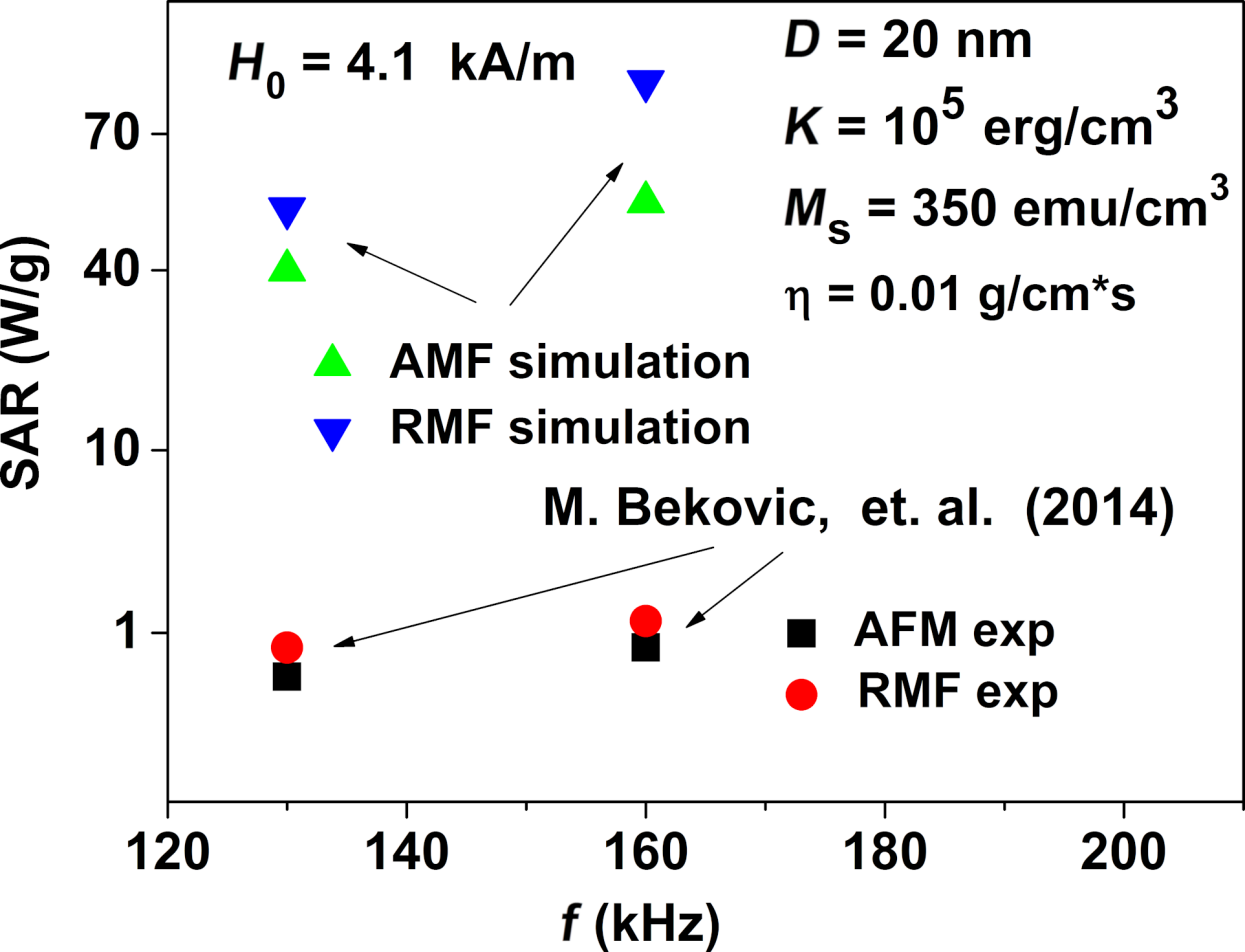
Comparison of the experimentally measured SAR values [41] at frequencies *f* = 130 and 160 kHz and amplitude *H*_0_ = 4.1 kA/m, with the SAR calculated numerically for a dilute assembly of magnetic nanoparticles in AMFs and RMFs.

One can see in Figure 6 that the numerical simulations performed at the same RMF frequency and amplitude predict SAR values of one order of magnitude larger than those measured in [41]. However, it should be noted that the numerical calculations presented in Figure 6 are carried out for an assembly of nanoparticles with diameter *D* = 20 nm. This diameter is close to the optimal diameter for particles with typical magnetic parameters of iron oxide, that is, *M*_s_ = 350 emu/cm^3^, *K* = 10^5^ erg/cm^3^, ρ = 5 g/cm^3^. Therefore, one can assume that the small SAR values measured in [41] are related to the fact that the average particle diameter in this experiment was far from the optimal value, *D* ≈ 20–24 nm. On the other hand, the calculated SAR values in RMFs turned out to be slightly higher than the corresponding SAR values in AMFs, in agreement with the results obtained in [41,43].

The numerical results obtained show that in order to achieve sufficiently high SAR values in magnetic hyperthermia, great attention should be paid to the proper choice of magnetic and geometrical parameters of the nanoparticles, as well as the selection of the appropriate RMF frequency and amplitude. It is worth noting that the frequencies *f* = 130 and 160 kHz, as well as the amplitude *H*_0_ = 4.1 kA/m investigated in Figure 6 correspond to domain I in the diagram of Figure 2. Due to the moderate SAR values obtained numerically this set of parameters is not suitable for magnetic hyperthermia. In order to determine the optimal RMF frequency and amplitude we calculated the SAR at various points in the diagram of Figure 2, considering the weaker Brezovich criterion [50], *fH*_0_ ≤ 6.25 × 10^4^ kHz·Oe.

As Figure 2 shows, in the Brezovich domain there are the magneto-dynamic modes I and II of nanoparticle motion in RMFs. Regarding this, it is interesting to investigate which type of particle magneto-dynamics is preferable for the use in magnetic hyperthermia. To answer this question we performed SAR calculations for a dilute assembly of magnetic nanoparticles in RMFs in two characteristic cases: a) at a given frequency, *f* = 120 kHz, over a range of amplitudes *H*_0_ = 50–550 Oe, and b) at a given field amplitude, *H*_0_ = 120 Oe, over a frequency range *f* = 50–1050 kHz. The calculations were performed for dilute assemblies of iron oxide nanoparticles with characteristic diameters *D* = 16, 20 and 30 nm. The liquid viscosity was taken to be η = 0.01 g/(cm·s), the medium temperature being *T* = 300 K.

**Figure 7:**
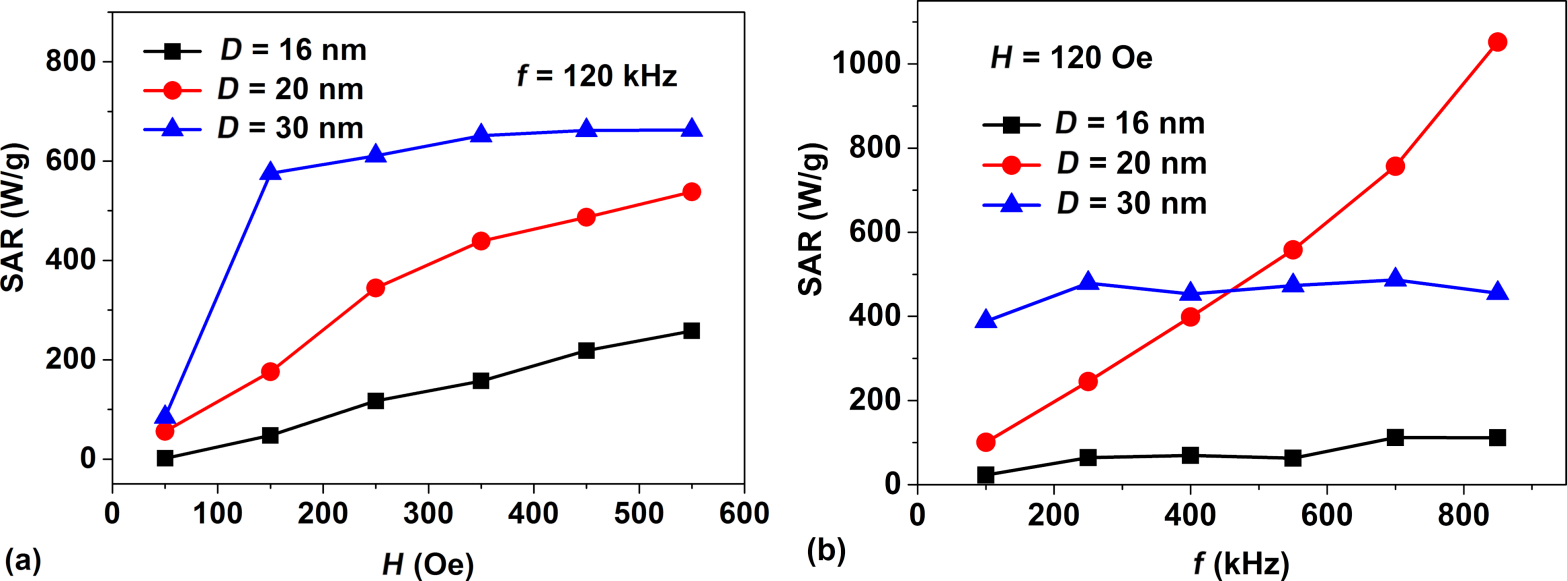
a) SAR of a dilute assembly of iron oxide nanoparticles of different diameters at a fixed frequency, *f* = 120 kHz, depending on the RMF amplitude; b) the same for a fixed RMF amplitude, *H*_0_ = 120 Oe, depending on the field frequency.

As Figure 7a shows, at a fixed frequency *f* = 120 kHz the SAR rises with increasing particle diameter or RMF amplitude. However, for nanoparticles of the maximum investigated diameter, *D* = 30 nm, the increase in SAR in the field interval *H*_0_ = 150–500 Oe is insignificant. Moreover, the use of variable magnetic fields of large amplitude requires the generation of strong electric currents, which may be unsafe in a medical clinic. In this regard, it seems more promising to use RMFs of moderate amplitude, *H*_0_ = 100–120 Oe, but at frequencies of about 400–600 kHz in magnetic hyperthermia. As Figure 7b shows, in this case SAR values of about 400–600 W/g can be obtained over a wide range of nanoparticle diameters, *D* = 20–30 nm.

## Conclusion

In this paper the dynamics of superparamagnetic nanoparticles in a viscous fluid in RMFs is studied using both numerical simulation and analytical calculations. This topic has recently attracted considerable interest [33–43] in view of the possibility of using magnetic nanoparticles in magnetic hyperthermia for the cancer treatment. Unfortunately, in experiments [41,43] very small SAR values, of the order of several watts per gram, have been measured in RMFs for an assembly of superparamagnetic nanoparticles distributed in a viscous liquid. However, the geometrical and magnetic parameters of the particles used in the experiments [41,43] were likely to be far from optimal values. Indeed, Figure 4 and Figure 5 show that for iron oxide nanoparticles very small SAR values should be observed if the particle diameter falls below 16 nm. In addition, RMFs of rather small amplitude have been used in these experiments.

Using numerical simulations we determined the optimal particle diameters, as well as the RMF frequencies and amplitudes at which the SAR of the assembly is large enough for application in magnetic hyperthermia. For a dilute assembly of superparamagnetic nanoparticles it is shown that at sufficiently high RMF frequencies there is an optimal range of particle diameters where the SAR of the assembly reaches maximum. For iron oxide nanoparticles of optimal diameters, *D* = 20–24 nm, the SAR in RMFs reaches values of 400–450 W/g at a frequency *f* = 400 kHz and a moderate amplitude *H*_0_ = 100 Oe. The theoretical SAR values obtained in RMFs exceed those in AMFs at the same frequency and field amplitude. It is important to note also that in the diameter range *D* > 24 nm the SAR in RMFs is approximately two times larger than that in AMFs. For magnetic hyperthermia in RMFs it is preferable to use magnetic fields of moderate amplitude, *H*_0_ = 100 Oe, in the frequency range of 400–600 kHz. In this case one can obtain SAR values of the order of 400–600 W/g over a wide range of particle diameters, *D* = 20–30 nm.

## Appendix

Based on the numerical results presented in Figure 1, the time dependence of the unit vectors 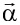 and 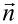 within the domains I and II in Figure 2 is assumed to be

[9]
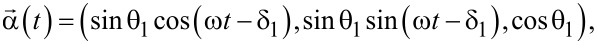


[10]
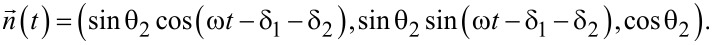


Here ω = 2π*f* is the given angular frequency of the RMFs in the *XY*-plane. The spherical angles θ_1_ and θ_2_ describe the deviation of the vectors 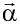 and 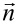 from the *XY*-plane. Angle δ_1_ gives a constant phase shift between the vector 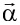 and the RMF vector (Equation 9), whereas angle δ_2_ gives a constant phase shift between the vectors 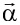 and 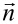. Thus, the unknown variables of the problem are four time-independent angles, θ_1_ and θ_2_, and δ_1_ and δ_2_.

First of all, it follows from Equation 9 and Equation 10 that the scalar product of unit vectors 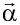 and 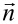 does not depend on the time:

[11]
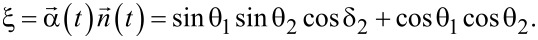


Substituting Equation 9 and Equation 10 into Equation 2, one finds that this equation is satisfied under the conditions

[12]
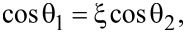


[13]
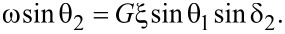


Similarly, it can be shown that Equation 9 and Equation 10 also satisfy Equation 11 provided that the following relations are fulfilled:

[14]
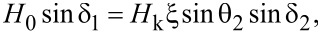


[15]
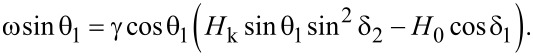


Thus, the unknown angles θ_1_, θ_2_, δ_1_, and δ_2_ are the solutions of the nonlinear set of Equations 11–15.

As Figure 2 shows, at a fixed RMF amplitude the first mode of motion exists at sufficiently low frequencies. It can be shown that if both unit vectors are in the same magnetic potential well, then their *z*-components are small and negative. Further we restrict ourselves to this case. The case when the unit vectors lie in opposite magnetic potential wells differs only in the signs of their *z*-components.

For the first mode of particle motion, the set of nonlinear Equations 11–15 can be analyzed in the limit of relatively small frequencies. Let us introduce a small parameter χ = ω/*G*. In the limit χ ≪ 1 the Equations 11–15 have the following solutions:

[16]
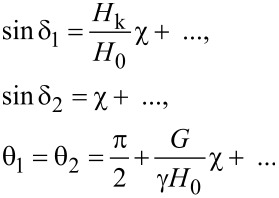


Equation 16 confirms that *z*-components of the vectors 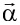 and 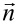 are small and negative. This is characteristic of the regime of stationary particle motion in the domain I in Figure 2.

For the second mode of particle motion, the *z*-components of the unit vectors are positive (if the vectors belong to the same magnetic potential well) and are of the order of unity. This case is realized at *H*_0_ < *H*_k_ with increasing frequency, ω/*G* ≈ 1, ω/*G* < 1. If the angles θ_1_ and θ_2_ are small, the set of Equations 11–15 allows for a solution:

[17]
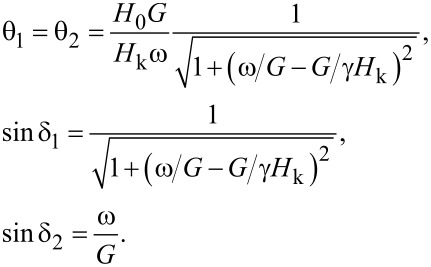


It is remarkable that the set of Equations 11–15 also makes it possible to estimate analytically the boundaries between the domains I–III for various stable modes of particle motion shown in Figure 2. Note that Equations 11–13 do not contain the angle δ_1_. If we eliminate the variable ξ from Equation 12 and Equation 13 using Equation 11, then we arrive at the relations

[18]
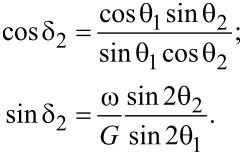


Using the basic trigonometric identity, one can express the angle θ_1_ through the angle θ_2_

[19]
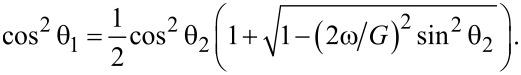


Further, using Equation 13, one can eliminate the variable ξ from Equation 16 and obtain the equation

[20]
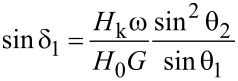


Thus, the angles θ_1_, δ_1_, and δ_2_ can be considered as functions of the angle θ_2_. Finally, for the angle θ_2_ one obtains from Equation 15 the equation

[21]
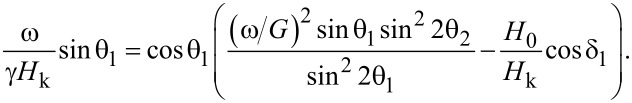


Equation 21 has a solution for cosθ_2_ < 0, θ_2_ ≈ π/2, which corresponds to the domain I in Figure 2. In addition, it also has a solution for cosθ_2_ > 0, which corresponds to domain II in Figure 2. Analysis of the solutions of the Equation 21 for the first and second magneto-dynamic modes of particle motion makes it possible to establish the domains of the existence of these modes on the plane (*f*, *H*_0_). The latter are shown in Figure 3.

## Supporting Information

Basic equations for the vectors **α** and **n** in magneto-dynamics (MD) approximation are shown in slide 2. Numerical simulation results for the dynamics of vectors **α** and **n** in different modes are given in slides 3–5. The dynamics of vectors **α** and **n** for modes 1, 2, and 3 are shown in slides 3, 4, and 5, respectively.

File 1 Basic equations for the vectors **α** and **n** in the magneto-dynamics (MD) approximation and numerical results of three different modes of unit magnetization vector.
