# Supplementary material for: Dynamics of superparamagnetic nanoparticles in viscous liquids in rotating magnetic fields
Source: Beilstein J Nanotechnol. 2019 Nov 22;10:2294–303. doi: 10.3762/bjnano.10.221 (PMC6880845; doi:10.3762/bjnano.10.221)
Supplement: File 1 — Basic equations for the vectors α and n in the magneto-dynamics (MD) approximation and numerical results of three different modes of unit magnetization vector. [file Beilstein_J_Nanotechnol-10-2294-s001.pptx]

## Slide 1
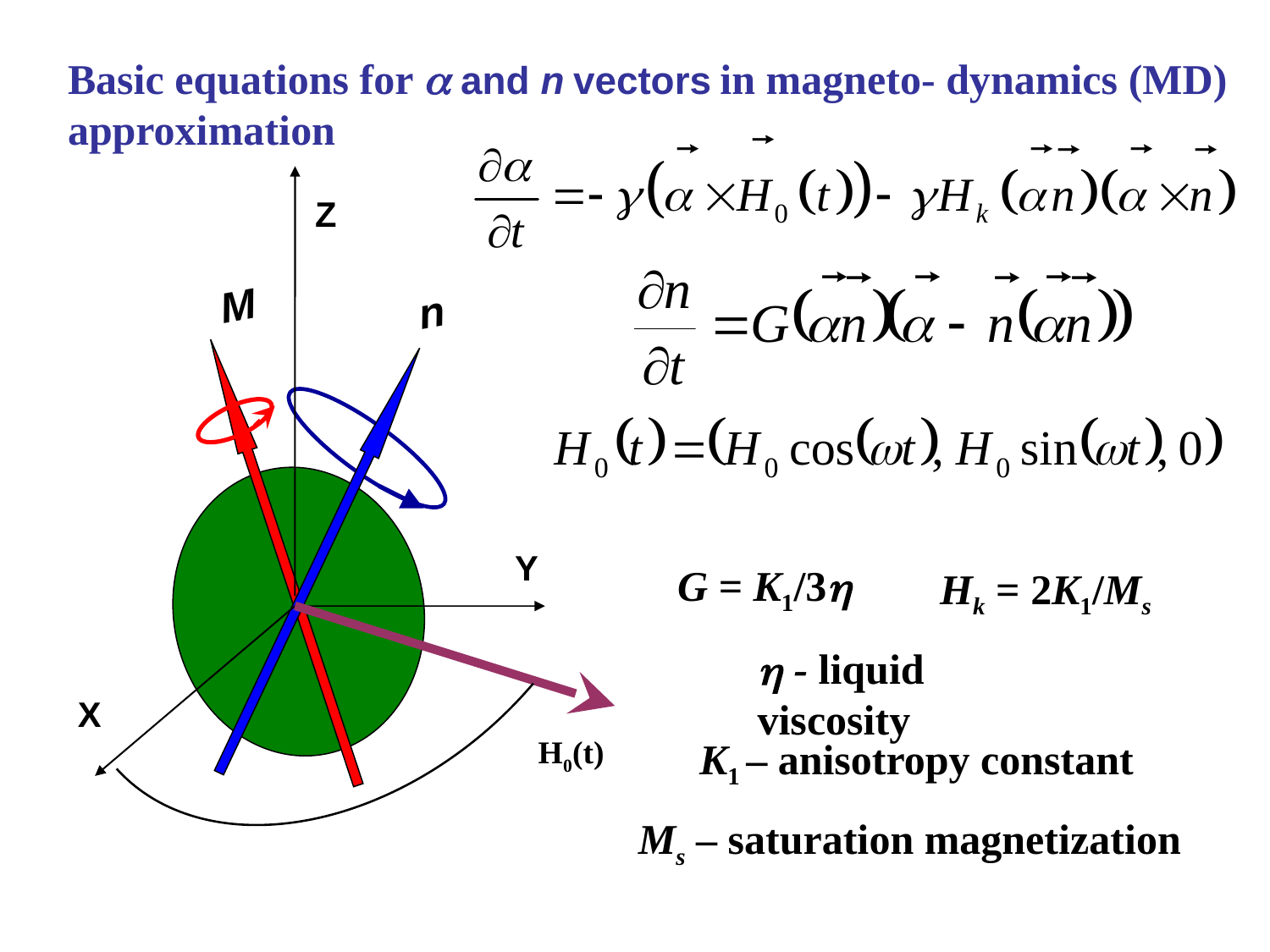

Basic equations for α and n vectors in magneto- dynamics (MD)
approximation
Z
M
n
Y
X
H0(t)
G = K1/3
Hk = 2K1/Ms
 - liquid viscosity
K1 – anisotropy constant
Ms – saturation magnetization

## Slide 2
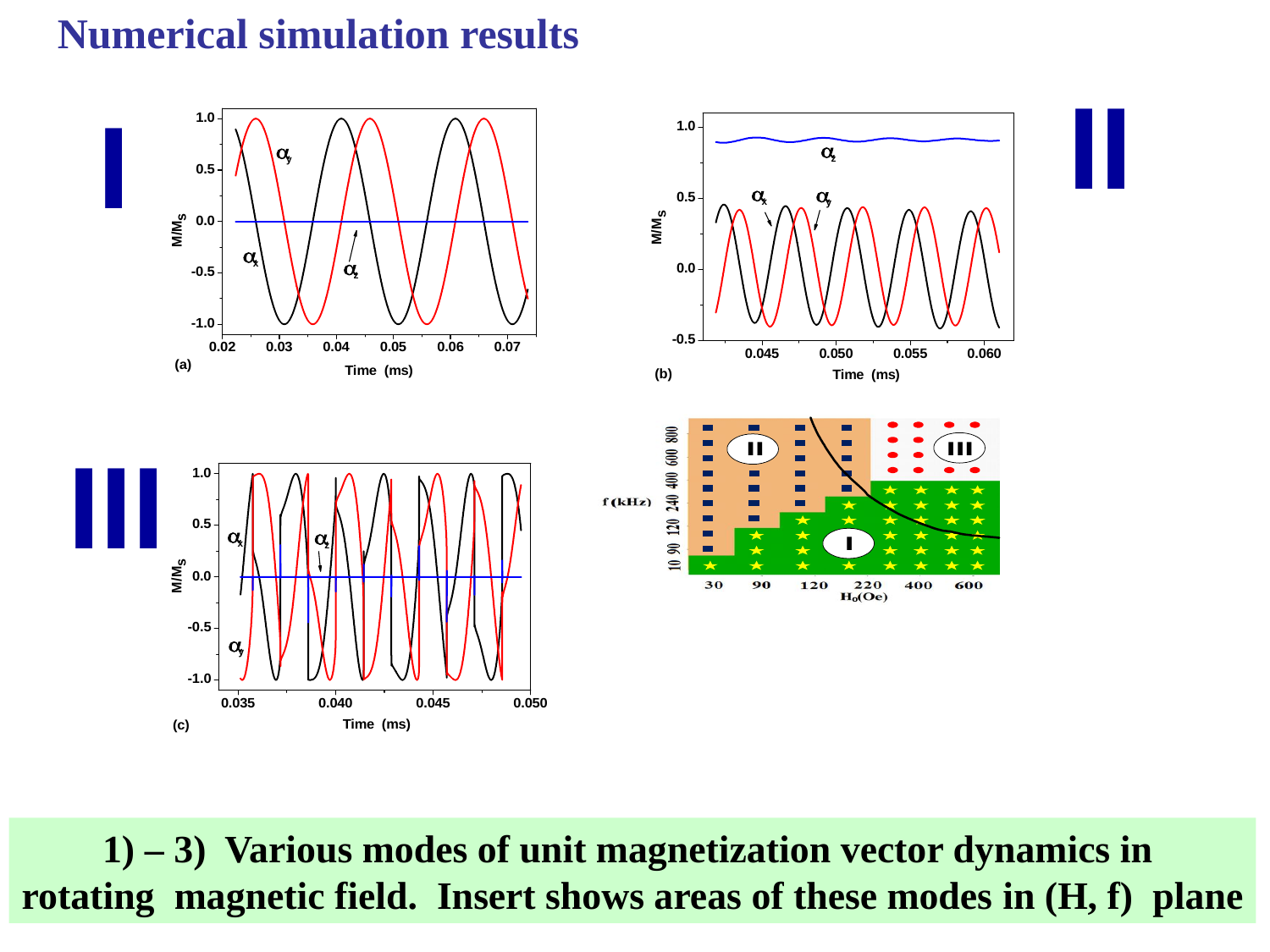

Numerical simulation results
II
I
III
1
2
3
1) – 3) Various modes of unit magnetization vector dynamics in
rotating magnetic field. Insert shows areas of these modes in (H, f) plane

## Slide 3
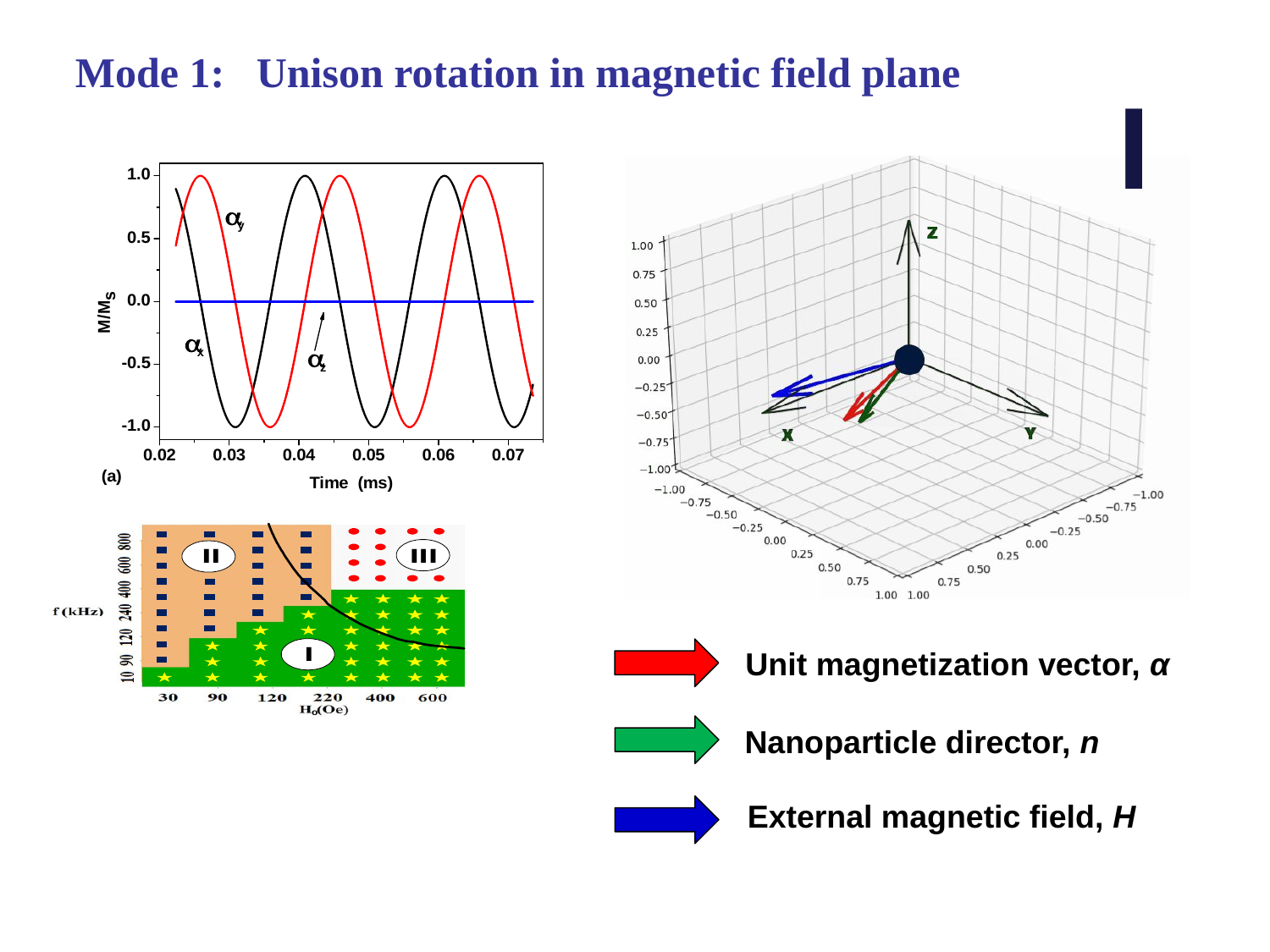

Mode 1: Unison rotation in magnetic field plane
I
Unit magnetization vector, α
Nanoparticle director, n
External magnetic field, H

## Slide 4
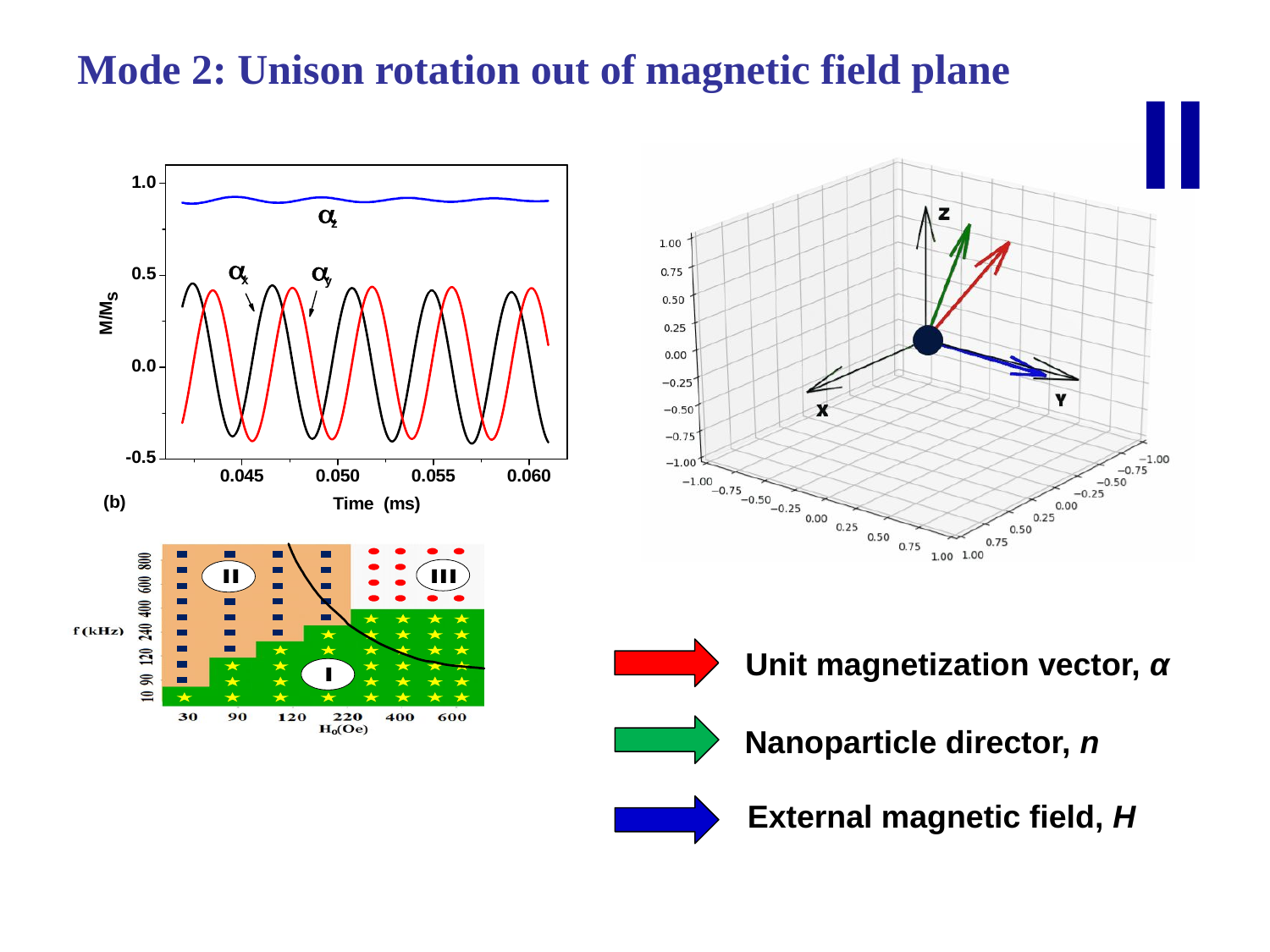

Mode 2: Unison rotation out of magnetic field plane
II
Unit magnetization vector, α
Nanoparticle director, n
External magnetic field, H

## Slide 5
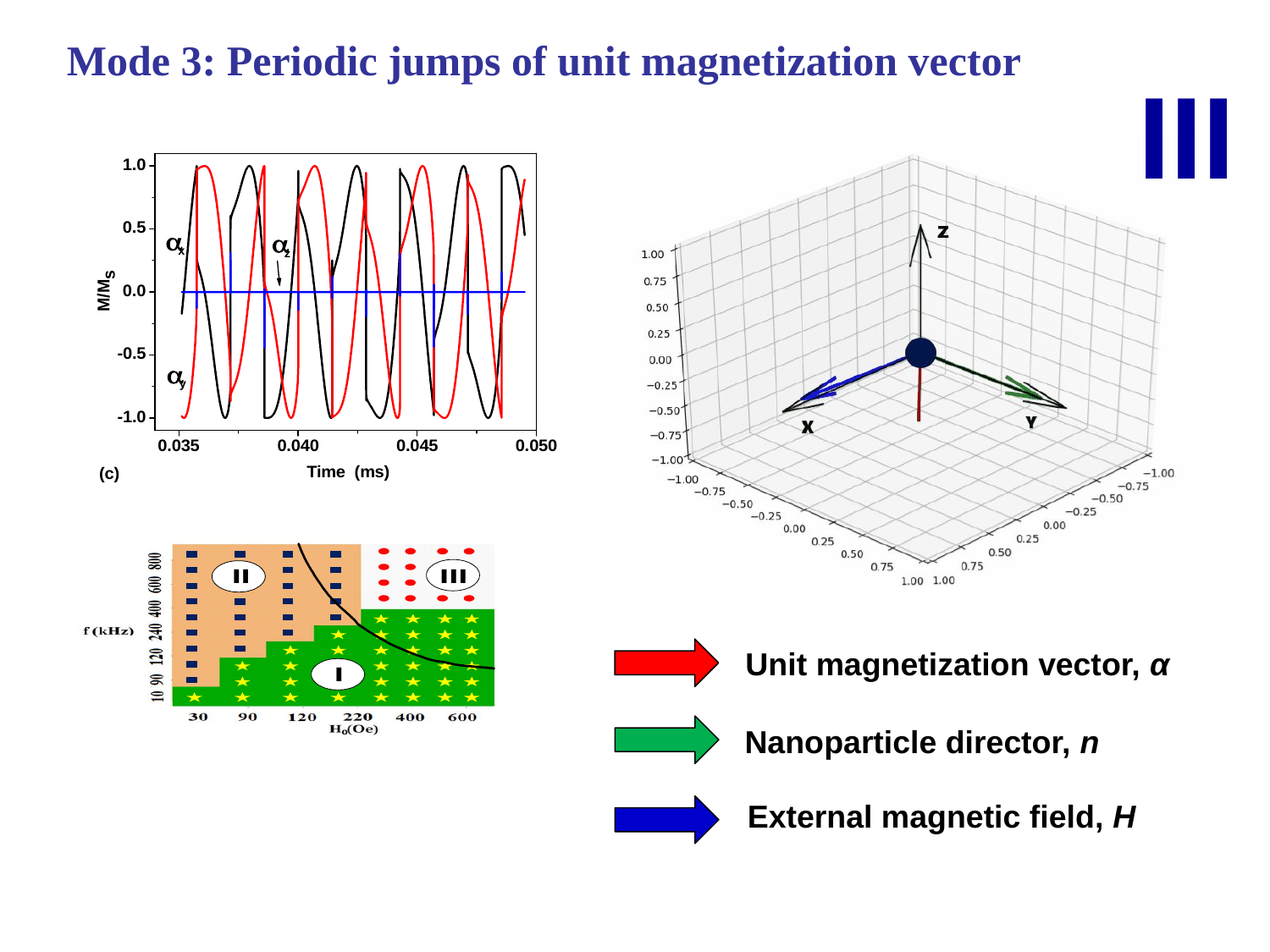

Mode 3: Periodic jumps of unit magnetization vector
III
Unit magnetization vector, α
Nanoparticle director, n
External magnetic field, H
